# Influence of interoception and body movement on the rubber hand illusion

**DOI:** 10.3389/fpsyg.2024.1458726

**Published:** 2024-12-11

**Authors:** Yoshitaka Kaneno, Achille Pasqualotto, Hiroshi Ashida

**Affiliations:** ^1^Graduate School of Letters, Kyoto University, Kyoto, Japan; ^2^Institute of Human Sciences, University of Tsukuba, Tsukuba, Japan

**Keywords:** rubber hand illusion, interoception, body movement, sense of body ownership, sense of agency

## Abstract

Rubber hand illusion (RHI) refers to the illusory sense of body ownership of a fake hand, which is induced by synchronous visuotactile stimulation to the real and fake hands. A negative correlation was reported between the cardiac interoception and the strength of RHI, but the subsequent studies have been unsuccessful in replicating it. On the other hand, voluntary action is suggested to link interoception and the sense of body ownership in different situations. If so, moving RHI, induced by the active or the passive finger tapping while observing a fake hand, might reveal the relationship more clearly. The measurement of interoception has been another issue. We, therefore, examined the relationship between the moving RHI and two measures of interoception: interoceptive accuracy (IAcc) measured by the conventional heartbeat counting task and interoceptive sensibility (IS) measured using a questionnaire. For the classical visuotactile RHI, our results supported the lack of association between the interoception measures and RHI. For the moving RHI, a stronger sense of body ownership was induced for participants with higher IS regardless of active or passive movement, and a stronger sense of agency was caused by active than passive movement only for those with lower IAcc. These results reveal the dynamic links between the interoception and the bodily senses. The results also suggest that multiple dimensions of interoception affect the bodily senses differently.

## 1 Introduction

The sense of body ownership (SBO), together with the sense of agency (SA), represents the “minimal self” (Gallagher, 2000) of our consciousness. The rubber hand illusion (RHI) (Botvinick and Cohen, 1998) has often been used as a tool in experimental studies on SBO. RHI refers to the illusory SBO over a fake rubber hand when the real and fake hands are synchronously stroked with a brush while only the rubber hand is visible. The RHI reveals that SBO is versatile. For example, it can even be extended to two-dimensional drawing (Pasqualotto and Proulx, 2015). Moreover, the “classical” RHI by brush strokes induces an illusory shift in the proprioceptive senses of the hand position [proprioceptive drift (PD)].

We have previously found that facial feedback could modulate RHI (Kaneno and Ashida, 2023), showing enhanced PD with negative emotional expressions. This effect, however, could have been mediated by interoceptive stimulation through the facial feedback procedure rather than the emotional state itself. Whether interoception is associated with RHI has been a matter of debate. Tsakiris et al. (2011) reported that RHI—both in terms of PD and subjective rating—negatively correlated with participants’ ability to monitor their own heartbeats. They suggested an active role of interoception in body-related multisensory integration. Later studies, however, have been unsuccessful in replicating the result, showing no association between cardiac interoception and RHI (measured by both questionnaire rating or PD): (Critchley et al., 2021; Crucianelli et al., 2018; Dobrushina et al., 2021; Horváth et al., 2020; questionnaire: Yamagata et al., 2023). Even a positive correlation was found with PD (Suzuki et al., 2013). We should note, however, some conditions were different, such as the use of virtual hands and the definition of interoceptive sensitivity by the heartbeat feedback judgment task.

A recent study by Ma et al. (2023) demonstrated that the relationship between interoception and the sense of embodiment in a virtual reality environment can be modulated by voluntary movement. They found that accuracy in the heartbeat counting task positively correlated with the whole-body walking drift toward the avatar when the task involved voluntary walking movement, suggesting that SA was a contributing factor. As to body movement, one problem with the classical RHI is that passive stimulation on a stationary hand does not evoke a strong SA. Kalckert and Ehrsson (2012), therefore, introduced a “moving RHI” method in which participants carry out tapping movement of their unseen finger while observing the synchronous or asynchronous movement of the rubber hand’s finger. The moving RHI might tell us more about the relationship among SBO, SA, and interoception, but there has been no such report.

Another problem lies in the equivocal aspects of interoception. Garfinkel et al. (2015) proposed three dimensions of interoception. First, *interoceptive accuracy* (IAcc) refers to cardioceptive accuracy measured by the heartbeat counting task (Schandry, 1981). Second, *interoceptive sensibility* (IS) is measured using questionnaires, such as the Body Perception Questionnaire (BPQ; Porges, 1993), which assesses multiple aspects of interoception, including feelings about dry mouth, breathing, swelling, and stomach statuses, in addition to cardiac sensation. Third, *interoceptive awareness* (IAwa) refers to metacognitive awareness of IAcc, which is not considered in this article because IAwa required additional experiments (heartbeat discrimination and/or more trials of heartbeat counting). Teraoka et al. (2023) found that IS, but not IAcc, positively correlated with illusory PD after participants looked at their hand’s image on the screen. The results by Ma et al. (2023) and Teraoka et al. (2023) consistently reported a positive correlation between interoception and body drift with active movement. Still, they were inconsistent as to the specific dimension of interoception.

This study aimed to explore the relationships between the bodily senses (SBO and SA) and interoception (IAcc and IS) under the classical and moving RHI conditions, with an expectation that active movement can reveal the relationship between SBO/SA and interoception.

## 2 Methods

### 2.1 Participants

Forty healthy students at Kyoto University volunteered with written informed consent and were compensated with a prepaid book card at the standard rate of Kyoto University. All had normal or corrected-to-normal vision and had no prior experience with RHI experiments.

The classical and moving RHIs (see Section 2.2.2) were tested for separate groups of participants to reduce the burden of each participant for better concentration and to deal with different factor designs (see Section 2.2.2). An *a priori* power analysis with G*Power 3.1.9.6 (Faul et al., 2007) for analysis of variance (ANOVA) (repeated measures; within–between interaction) required 20 participants for the moving RHI condition, assuming a medium effect size (*η*_p_^2^ = 0.1), power of 0.8, correlation among repeated measures of 0.5, and *α* of 0.05. This number of participants was also applied to the classical RHI condition. Therefore, 20 participants (4 females and 16 males, age = 19–27 years) were randomly assigned to the classical RHI condition, and the other 20 (7 females and 13 males, age = 19–34 years) were assigned to the moving RHI condition.

### 2.2 Apparatus and stimuli

The experiments were conducted under room illumination (~400 lux) with white light-emitting diode (LED) light.

#### 2.2.1 Interoception

For IAcc, used a conventional heartbeat counting task for IAcc (Schandry, 1981). We monitored participants’ heartbeats using a pulse sensor kit (World Famous Electronics LLC; https://pulsesensor.com/) that measured the blood flow optically on the tip of the participant’s left index finger. The sensor was connected to an Arduino Uno R3 (Arduino) microcomputer that counted the pulses online. The pulse waves were also recorded on a laptop personal computer (PC) running Windows 10 (Microsoft Corp., Redmond, WA, USA), and the pulse counts were visually checked later.

For IS, we used the Japanese version of the Body Perception Questionnaire Very Short Form (BPQ-BA-VSF-J), which was translated from Table 2 of the study by Cabrera et al. (2018) by Kobayashi et al. (2021) (shown in Supplementary Table S1 with permission). The participants responded to the 12 questionnaire items on how much they were aware of various aspects of interoception using a 5-point Likert scale [from 0 (not at all) to 4 (always)]. The laptop PC was used for collecting the responses of the participants.

#### 2.2.2 Rubber hand experiments

**Figure 1:**
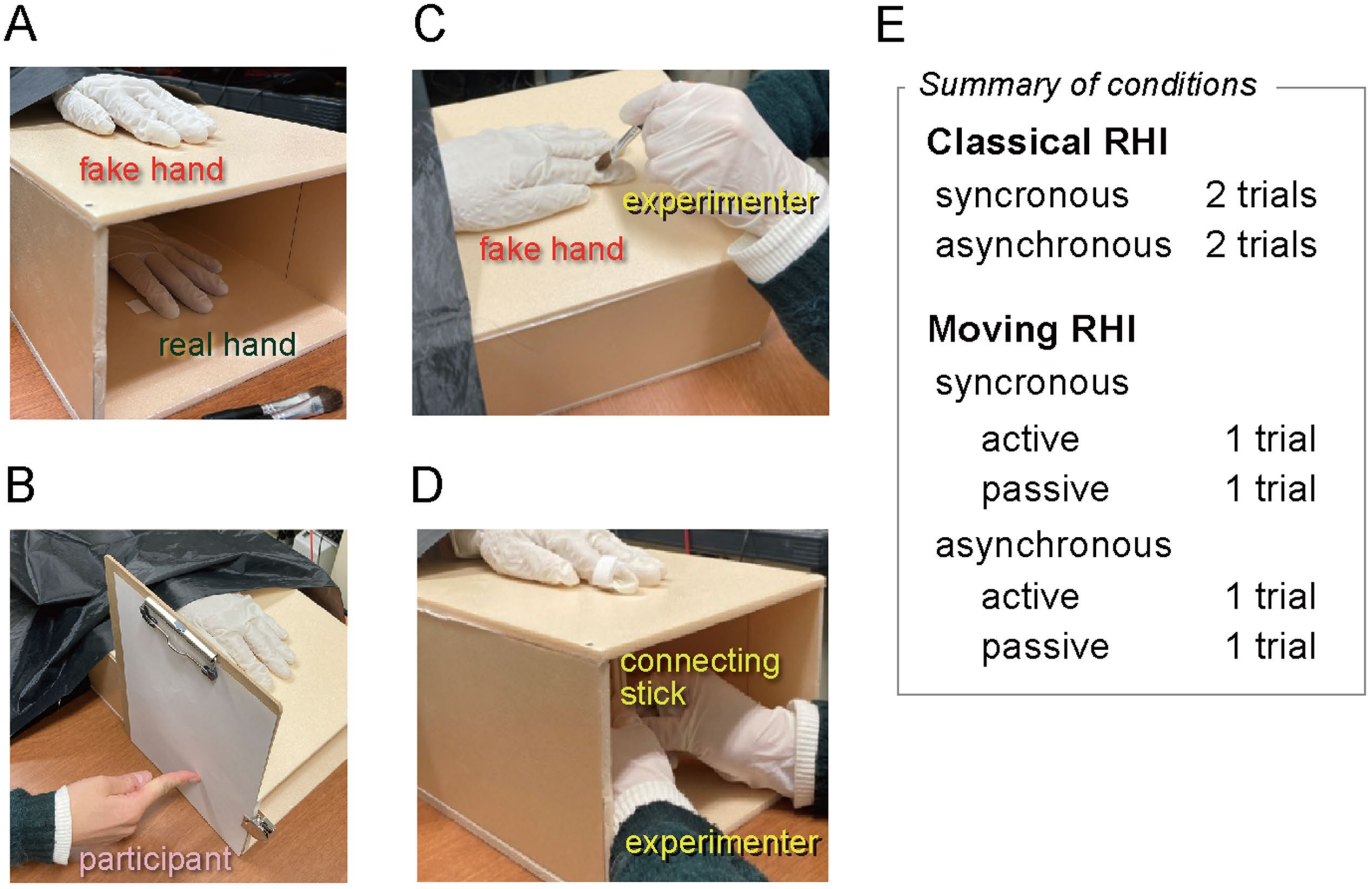
The setup and conditions of RHI experiments. **(A)** The fake hand was placed on top of the box, while the participant’s hand was placed inside and below. **(B)** The paper clipboard for measuring the proprioceptive drift (removed in the other pictures for clarity.) **(C)** The classical RHI stimulation with a paintbrush. **(D)** The moving RHI stimulation. The connecting stick was adjusted in each experimental block so that the participant or the experimenter moved the fake hand’s finger to be synchronous or asynchronous with the participant’s finger. A summary of trial conditions is shown to the right of parts **(C)** and **(D)**.

The apparatus was built following Kalckert and Ehrsson (2012), Kalckert and Ehrsson (2014a), and Kalckert and Ehrsson (2014b), and the procedures essentially followed them. A silicon left hand was placed on a polystyrene foam box (w250 × h180 × d320 mm) with both ends open (Figure 1A). The height of 18 cm was chosen to compromise the ease of the experimenter’s handling action. Participants were seated in front of the box and placed their left hand inside it, right under the silicon hand. They wore a black cape so they could see neither their hand nor the back end of the silicon hand. Both the silicon and real hands were covered with the same white rubber gloves to prevent direct touch by the experimenter and minimize any differences in appearance. For PD measurements, a paper clipboard was attached to the side of the box, so that the participant could point to the perceived position of the hidden left index finger with the right index finger (Figure 1B).

For the classical RHI, the fake and the real hands were gently brushed by the experimenter around the distal phalanx of the index finger (Figure 1C) at approximately 1 Hz, either synchronously (i.e., both real and fake fingers at the same time) or asynchronously (i.e., in antiphase to each other by brushing the fake finger with a delay of approximately 0.5 s). The brushing location was limited to minimize the difference from the moving RHI condition.

For the moving RHI, a wooden stick linked the index fingers of the real and the fake hands around the distal phalanx so that the fake finger moved synchronously with the real finger (Figure 1D; see also Kalckert and Ehrsson, 2014a, Figure 1). The participant performed finger tapping at approximately 1 Hz in the *active* trials, while in the *passive* trials, the experimenter moved the participant’s finger similarly. The participant’s finger moved the fake finger via the linking stick in the *synchronous* trials (either actively or passively), while the link was detached in the *asynchronous* trials, and the experimenter moved the fake finger roughly in the antiphase to the real finger (see Supplementary videos S1–S4). Participants practiced tapping with a metronome before the experiment. There were no guiding signals in the actual trials.

Subjective RHI was assessed using a questionnaire consisting of eight items, which were translated from the English statements used by Berger et al. (2023, Table 1) into Japanese by the first author (YK) (see Supplementary Table S2). Items 1 and 2 were for measuring SBO, and items 3 and 4 were control statements. Items 5 and 6 were for SA, and items 7 and 8 were for controls. Participants responded using a 7-point Likert scale from −3 (strongly disagree) to 3 (strongly agree).

### 2.3 Procedure

After reporting demographic information such as their sex and age, participants were tested for interoception before the RHI tasks, following Tsakiris et al. (2011). They answered the questionnaire (BPQ-BA-VSF-J) for IS and handedness questionnaire (Okubo et al., 2014) using a laptop PC. Sex, age, and handedness were not used in the later analyses because the number of participants was not balanced. Then, for IAcc, they performed the heartbeat counting task 3 times. They put on the pulse sensor and rested for at least 100 s while the experimenter checked the sensor outputs. Participants were asked to count the number of their heartbeats during the period between two auditory tones. In random order, the trial durations were 25, 35, and 45 s. In each trial, participants reported the number and the confidence on a 7-point scale. Confidence was not analyzed in this study.

Then, the main RHI experiment was conducted. According to Berger et al. (2023), the duration of the stimulation was 60 s under all the conditions. For the classical RHI, two synchronous and two asynchronous trials were tested randomly. For the moving RHI, each of the four conditions (active/passive × synchronous/asynchronous) was tested once. Synchronous and asynchronous trials were tested in separate blocks to minimize adjustment to the linking stick. The trial order and the block order were counterbalanced across participants.

Before and after each RHI stimulation, for measuring PD, the participants were asked to point to their left index finger using their right index finger on the right side of the box, and the experimenter marked the position on the clipboard paper (Figure 1B). The participants could not see the marks. At the end of each trial, the participants answered the RHI questionnaire.

The whole experiment took approximately 50 min.

### 2.4 Analyses

We used R version 4.4.0(R Core Team, 2024, RRID:SCR_001905) and RStudio version 2024.04.1 (RStudio Core Team, 2024, RRID:SCR_000432) for the analyses. We applied separate statistical analyses to classical and moving RHIs for the different designs and the different pools of participants. Holm–Bonferroni correction of *p*-values was applied when tests were repeated within each set of analyses. The Shapiro–Wilk tests were used for testing normality. For ANOVA tests, Mendoza’s multisample sphericity test was conducted, and Greenhouse–Geisser correction was applied when sphericity was violated. We used Cohen’s *d* and partial *η*^2^(*η_p_*^2^) to estimate the effect size.

The IAcc score for each participant was calculated in a conventional way as 1 − |*H*_rec_ − *H*_rep_|/*H*_rec_ for each trial, averaged across the three trials. *H*_rec_ is the number of recorded heartbeats, and *H*_rep_ is the reported heartbeat count. IAcc score takes a value of 1 or less, with a larger value indicating more accurate counts (e.g., Filippetti and Tsakiris, 2017). The IS score for each participant was defined as the sum of the 12 scores of BPQ-BA-VSF-J. Pearson’s correlation coefficient (*r*) between IAcc and IS scores was computed.

For each item of the RHI questionnaire, scores from synchronous and asynchronous stimulations were compared under each RHI condition. The scores from the two trials were averaged before performing subsequent analyses for classical RHI. Wilcoxon’s signed-rank test was used due to the typical non-normal distribution of the data and the discrete responding scale. The SBO index was defined as the mean of synchronous–asynchronous scores of items 1 and 2. SA index was similarly computed from the scores of items 5 and 6.

The PD score in each trial was calculated as the difference between the positions reported before and after each RHI stimulation, with a positive score indicating PD toward the fake hand after the stimulation. The PD scores were compared between synchronous and asynchronous conditions using paired-sample *t*-tests for classical RHI and two-way repeated-measures ANOVA for moving RHI. The PD index was defined as synchronous–asynchronous PD scores.

If necessary, Pearson’s correlation coefficients (*r*) and partial correlations were computed among the SBO, SA, and PD indices. If necessary, a *post-hoc* comparison of the correlations between the active and passive moving RHI conditions was conducted using the Cocor package v1.1.4 on R (Diedenhofen and Musch, 2015).

For IAcc and IS, the participants were median-split into high-and low-interoceptive groups (following Tsakiris et al., 2011). The SBO, SA, and PD indices were then compared between the two groups. Paired-samples *t*-tests were used for classic RHI, and two-way mixed-design ANOVAs were conducted for moving RHI, with the factors of the interoceptive group (high-IAcc/low-IAcc or high-IS/low-IS) and the movement type (active/passive).

## 3 Results

### 3.1 Interoception measures

**Figure 2:**
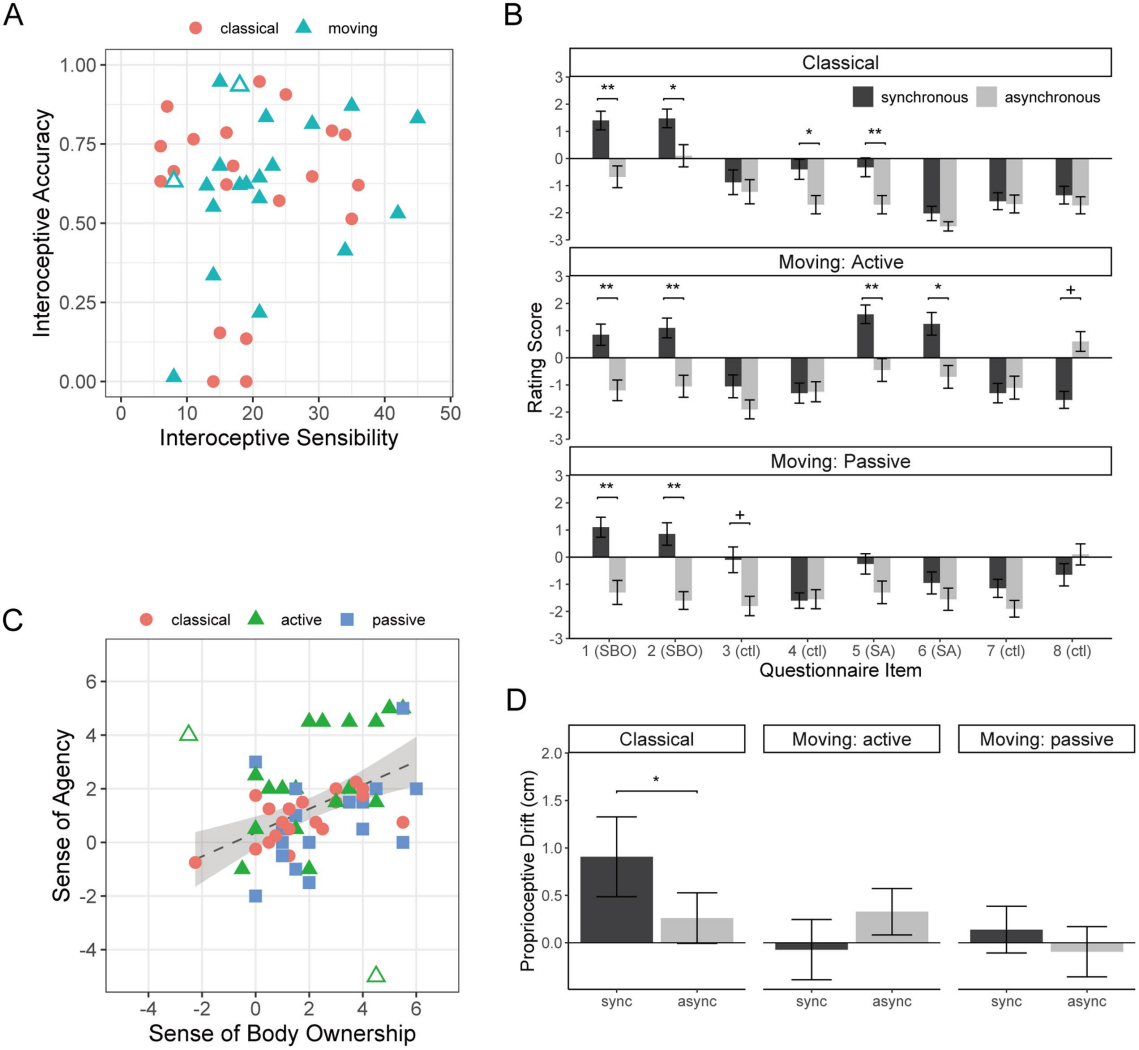
**(A)** The results of interoceptive measures of IS and IAcc are shown as a scatter plot. Each marker represents a single participant. The dashed line shows the linear regression line, with the gray shades showing the 95% confidence intervals. The outlined symbols represent the outliers that were to be excluded in the later analyses. **(B)** The plot of rating scores under the three RHI conditions is plotted separately for synchronous and asynchronous stimulation. The error bars show standard errors of the mean across participants. SBO, sense of ownership, SA, sense of agency, ctl, control. *p < 0.05; **p < 0.01; ***p < 0.001. **(C)** A scatter plot of SBO and SA. Each marker represents a participant, but a participant appears twice for the active and passive conditions of moving RHI. The outlined symbols show the outliers that were excluded in the analyses. The dashed line shows the linear regression line (without the outliers) with the gray shades showing the 95% confidence intervals. **(D)** A plot of proprioceptive drift (PD) for synchronous and asynchronous stimulations for each RHI condition.

Figure 2A shows the IAcc and IS scores as a scatter plot. The correlation between them was weak and insignificant (*r* = 0.17, *p* = 0.31), supporting the separation of these dimensions of interoception (Garfinkel et al., 2015). Descriptive statistics are shown in Supplementary Tables S3, S4.

### 3.2 Senses of body ownership and agency from the questionnaire

Item-wise averages of rating scores are plotted in Figure 2B for each RHI condition, separately for synchronous and asynchronous trials. The Shapiro–Wilk test revealed deviation from normality for 44 out of 48 combinations (3 RHI types × 2 synchronous/asynchronous × 8 questionnaire items) (*Ws* < 0.901, *ps* < 0.042), supporting the use of a non-parametric test. Supplementary Table S5 details synchronous tests vs. asynchronous tests. As to the SBO (items 1 and 2), rating scores were significantly higher in the synchronous trials than in the asynchronous trials for all RHI types, with all synchronous scores being positive. The control item 4 yielded a significant difference for the classical RHI, but the rating scores were negative. Illusory SBO was, therefore, successfully induced by the synchronous stimulation both for classical and moving RHI. As to SA (items 5 and 6), the scores were significantly higher in the synchronous trials than in the asynchronous scores for actively moving RHI, with positive synchronous scores. Item 5 also yielded a significant difference for classical RHI, but the scores were negative. Therefore, the illusory SA was successfully induced by active finger movement with synchronous visual information.

The scores of the majority of the control items were below zero, confirming the task compliance of the participants (Kalckert and Ehrsson, 2012; Berger et al., 2023). One exception was the asynchronous score of item 8, which was positive and higher than the synchronous score, although the difference was not significant (*p* = 0.067). This pattern of result was similar to that in Berger et al. (2023, Figure 3A), which can be understood by the movement of the rubber finger against active control.

**Figure 3:**
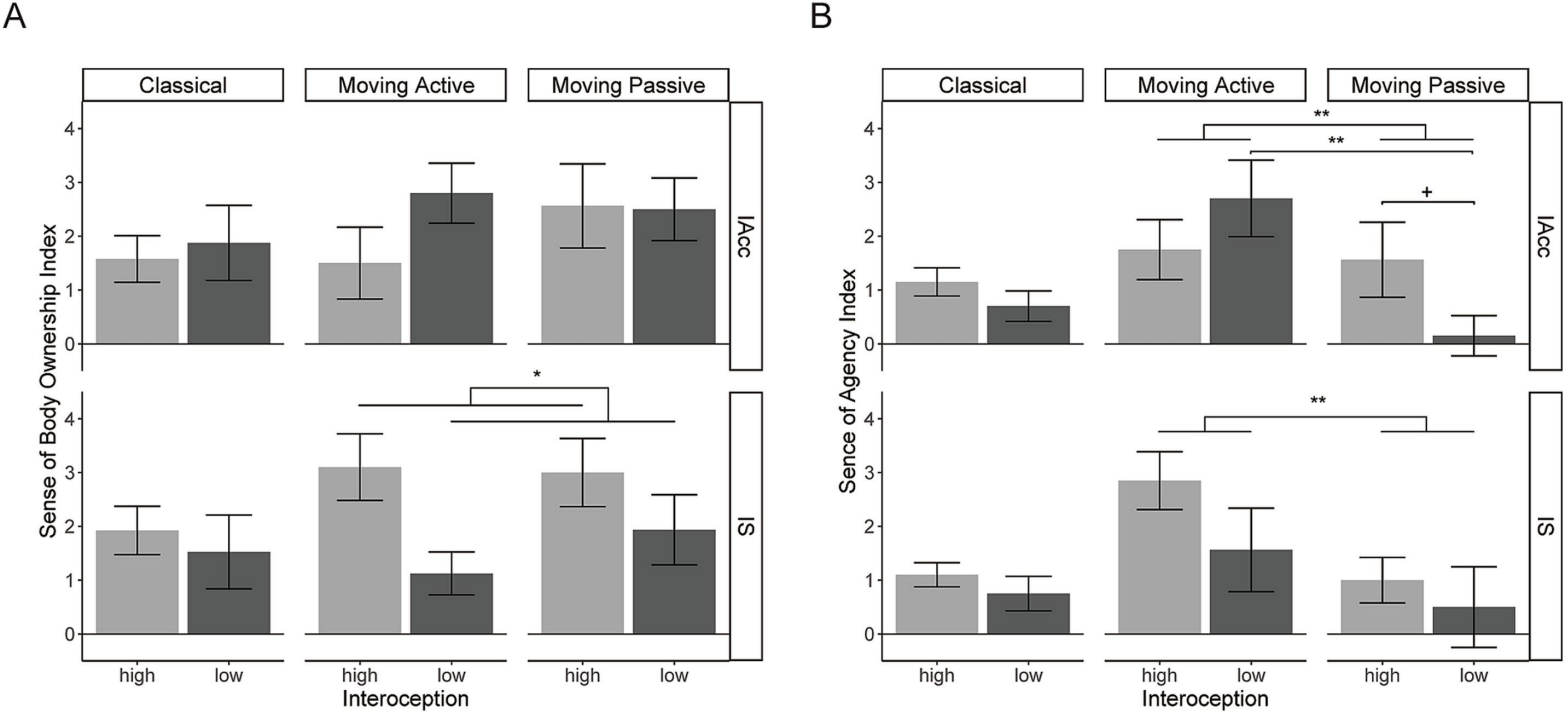
**(A)** The plot of the SBO index for high and low IAcc or IS for each RHI condition. **(B)** The plot of the SA index is plotted in the same way as the SBO index. Two outliers (see text) were excluded in these plots. The error bars show standard errors of the mean across participants. +, *p* < 0.1; *p < 0.05; **p < 0.01.

Figure 2C shows a scatterplot of SBO and SA indices computed from the questionnaire responses. There was a “moderate” (Evans, 1996) positive correlation between them for the classical RHI (*r* = 0.58, *p* = 0.015). There are two obvious outliers in the active-moving condition as indicated by the outlined markers, which were objectively marked as anomalies using the isolation forest method (Liu et al., 2008; see Supplementary Figure S1 for details). Removing these two participants yielded a “strong” correlation for the active condition (*r* = 0.65, *p* = 0.012), and a “moderate” correlation for the passive condition (*r* = 0.47, *p* = 0.049). The difference in *r* between active and passive conditions was insignificant (*p* > 0.43). We excluded the data from the two outliers in all the analyses, discussed in the following subsections.

The descriptive statistics of SBO and SA questionnaire items are summarized in Table 1. Synchronous stimulation induced SBO (median ≥ 1) in all three RHI types and SA only in the active movement condition. Statistical tests were performed only on each questionnaire item to reduce repetitions of tests on the same set of data. The Shapiro–Wilk test did not show significant deviation from normality in the SBO and SA indices (*Ws* > 0.905, all *ps* > 0.070), except for one condition (SBO for passive RHI, *W* = 0.896, *p* = 0.048, not corrected). We will use ANOVA in the interoception-based analyses, as ANOVA is generally robust to violation of normality (e.g., Schmider et al., 2010).

**Table 1:** Descriptive statistics of the questionnaire ratings, as in the format of Tables 2 and 3 provided in the study by Kalckert and Ehrsson (2014a).

	Synchronous				Asynchronous			
Quantiles	SBO	SBO control	SA	SA control	SBO	SBO control	SA	SA control
Classical								
Median	1.63	−1.13	−1.25	−1.25	0.00	−2.13	−2.50	−1.75
25%	1.00	−2.06	−2.06	−2.38	−1.75	−3.00	−3.00	−2.75
75%	2.56	1.06	−0.69	−0.94	1.06	0.06	−1.69	−1.00
Moving (active)								
Median	1.00	−1.00	2.00	−1.00	−1.50	−2.50	−1.00	−0.50
25%	0.50	−2.00	1.00	−2.50	−2.38	−2.50	−2.38	−1.50
75%	1.50	0.00	2.50	−0.50	−0.63	−1.00	0.88	1.13
Moving (passive)								
Median	1.00	−0.75	−0.75	−1.00	−2.00	−2.00	−2.00	−0.75
25%	0.00	−2.38	−1.88	−1.88	−2.88	−3.00	−2.50	−1.75
75%	2.38	0.38	0.00	−0.13	−1.00	−0.50	−0.50	−0.50

Illusory SBO (median ≥ 1) was induced under all three RHI types. Substantial SA was induced only under the actively moving condition. Control items for both SBO and SA did not yield positive median scores. The two outliers were excluded. Including the outliers did not essentially change the result (see Supplementary Table S3). SBO: sense of body ownership; SA: sense of agency.

### 3.3 Proprioceptive drift

Figure 2D shows the PD scores. The Shapiro–Wilk test did not show significant deviation from normality in the PD scores under any condition of Figure 2D (*Ws* > 0.91, *p* > 0.079). For the classical RHI, an expected pattern of larger drift in synchronous than asynchronous trials was found [*t*(19) = 2.34, *p* = 0.030, *d* = 0.358]. The PD index significantly correlated with the SBO index (*r* = 0.66, *p* = 0.0097), which is consistent with the literature (see Tosi et al., 2023, for review and meta-analyses). The PD index was also significantly correlated with the SA index (*r* = 0.65, *p* = 0.010); however, a large part of this should be indirect, as the partial correlation between the PD and SA indices adjusted for the SBO index was not significant (0.43, *p* = 0.063). For the moving RHI, the PD was nearly zero and the PD index was not correlated with either index of SBO (active: *r* = 0.081, *p* > 0.99; passive: *r* = 0.16, *p* > 0.99) or SA (active: *r* = 0.25, *p* = 0.93; passive: *r* = 0.31, *p* = 0.82).

### 3.4 RHI and interoception

#### 3.4.1 Body ownership and IAcc

The SBO index is plotted against the IAcc groups as shown in the upper part of Figure 3B. The classical RHI did not yield a significant difference between the high-IAcc and low-IAcc groups (*t*[18] = 0.37, *p* > 0.9, *d* = 0.16). The moving RHI also did not yield significant effects; two-way mixed-effect ANOVA showed no significant effects of the IAcc group [*F*(1,16) = 0.65, *p* = 0.43, *η_p_*^2^ = 0.039], movement type (passive/active) [*F*(1,16) = 0.61, *p* = 0.44, *η_p_*^2^ = 0.037], or their interaction [*F*(1,16) = 1.96, *p* = 0.18, *η_p_*^2^ = 0.109].

#### 3.4.2 Body ownership and IS

The SBO index is plotted against the IS groups as shown in the lower part of Figure 3B. The classical RHI did not yield a significant difference between the high-IS and low-IS groups [*t* (18) = 0.49, *p* > 0.9, *d* = 0.22]. The moving RHI yielded a significant main effect of the IS group [*F* (1,16) = 4.89, *p* = 0.042, *η_p_*^2^= 0.234], with a larger SBO index for the high-IS group, but no significant effects of movement type [*F* (1,16) = 0.50, *p* = 0.49, *η_p_*^2^ = 0.030] or their interaction [*F* (1,16) = 0.82, *p* = 0.38, *η_p_*^2^ = 0.049].

#### 3.4.3 Agency and IAcc

The SA index is plotted against the IAcc groups as shown in the upper part of Figure 3C. The classical RHI did not yield a significant difference between the high-IAcc and low-IAcc groups [*t*(18) = 1.17, *p =* 0.52, *d* = 0.52]. The moving RHI did not yield a significant main effect of the IAcc group [*F*(1,16) = 0.10, *p* = 0.75, *η_p_*^2^ = 0.0063], but it did not yield significant effects of movement type [*F*(1,16) = 9.57, *p* = 0.0070, *η_p_*^2^ = 0.37] and their interaction [*F*(1,16) = 7.13, *p* = 0.017, *η_p_*^2^ = 0.31]. The simple main effect of movement type was significant in the low-IAcc group [*F*(1,9) = 18.74, *p* = 0.0019, *η_p_*^2^ = 0.68], but not in the high-IAcc group [*F*(1,7) = 0.081, *p* = 0.78 *η_p_*^2^ = 0.011]. That is, larger SA was induced with active than passive finger movement only in the low-IAcc group. The simple main effect of the IAcc group for passive movement was marginal but not significant [*F*(1,16) = 3.57, *p* = 0.077, *η_p_*^2^ = 0.18]. Other simple main effects were not significant (*p*s > 0.3).

#### 3.4.4 Agency and IS

The SA index is plotted against the IS group as shown in the lower part of Figure 3C. The classical RHI did not yield a significant difference between the high-IS and low-IS groups [*t*(18) = 0.895, *p* = 0.52, *d* = 0.40]. The moving RHI yielded a significant effect of movement type [*F*(1,16) = 7.76, *p* = 0.013, *η_p_*^2^ = 0.33], revealing a higher SA index with active than passive movement. The effects of the IS group [*F*(1,16) = 1.66, *p* = 0.22, *η_p_*^2^ = 0.094] and their interaction [*F*(1,16) = 0.57, *p* = 0.46, *η_p_*^2^ = 0.034] were not significant.

#### 3.4.5 PD and IAcc/IS

For the classical RHI, the PD index was not modulated by either IAcc [*t*(18) = 0.742, *p* = 0.47, *d* = 0.16] or IS [*t*(18) = 1.59, *p* = 0.26, *d* = 0.71]. For the moving RHI, there were no significant effects of the IAcc/IS group, movement type, or interactions on the PD index (*ps* > 0.26; see Supplementary Table S8; Supplementary Figure S2).

## 4 Discussion

The questionnaire results revealed that an illusory sense of body ownership (SBO) was induced under both classical and moving RHI conditions during synchronous visuotactile or visuomotor stimulation, and that sense of agency (SA) was induced only under the active moving condition. Overall, the manipulation of RHI was successful. Measurement of proprioceptive drift failed in the moving RHI, but this could be explained by the distance between the real and the fake hands, which turned out suboptimal. Kalckert and Ehrsson (2014b) found significant PD at the distance of 12 cm, but not at 27.5 cm. Substantial SA but not the SBO was induced at 27.5 cm. The distance of 18 cm in our setting was in between, at which SBO as well as SA could be induced while PD was abolished. We, therefore, cannot discuss the PD results. We will discuss the association between interoception and RHI based on the questionnaire results.

For the classical RHI, we did not find a significant association between measured SBO and either IAcc or sensibility (IS), which is in line with the literature that failed to replicate negative correlation between RHI and interoception (Critchley et al., 2021; Crucianelli et al., 2018; Dobrushina et al., 2021; Horváth et al., 2020; Yamagata et al., 2023). As expected, SA was not strongly induced in the classical RHI and was not significantly affected by either IAcc or IS.

Our novel findings are twofold. First, induced SBO under moving RHI positively correlated with IS regardless of active or passive movement. This result suggests that interoceptive sensation, when coupled with proprioception of the moving finger, might modulate SBO induced by the synchronous visual movement. Such a relationship, however, was not observed with IAcc, which reflected heartbeat counts. These results are parallel to those in the study by Teraoka et al. (2023) who reported a positive correlation between hand drift and IS but not IAcc, with a different experimental manipulation (see the “Introduction” section). On the contrary, our result is inconsistent with Ma et al. (2023), which showed a positive correlation between IAcc and the illusory drift of the whole body. These results suggest that different interoception aspects should affect the senses of different body portions.

Second, we found a significant effect of active motion on the measured SA only in the low-IAcc group. If extroceptive, proprioceptive, and efferent signals contribute to SA, cardiac interoception might adjust the balance. Those with lower IAcc may have more weight on the motor efference, while those with higher IAcc might rely more on the proprioception (hence less affected by active movement). However, the underlying mechanism is open for further investigation. Our results, on the contrary, did not show a positive correlation between the measured SA and IAcc (Koreki et al., 2022; Stern et al., 2020). Such a correlation was suggested in the passive condition (not significant, although *p* = 0.077). The reason for this discrepancy is also open, but we should note that the SA was quantified differently. In Stern et al. (2020), participants made a binary judgment on the SA (yes/no) regardless of the strength. Koreki et al. (2022) used the intentional binding task that provided indirect measures of SA.

Our results supported separate IAcc and IS dimensions because IAcc modulated SA and IS modulated SBO. This also underpins the aim of moving RHI to dissociate SBO and SA. On the contrary, the correlation between the SBO and SA indices was higher with active motion, although the difference in *rs* was not significant. Visual inspection of Figure 3 also suggests a tighter coupling of SBO and SA with active motion, as it appears that SBO and SA were more similarly affected with active than passive movement. We did not plan this comparison *a priori*, and this point may require investigation with a more appropriate design and statistical power.

In summary, active movement in moving RHI revealed the relationships between interoception and the bodily senses of ownership and agency. The relationships, however, are not straightforward, as there are interactions with the separate dimensions in interoception (Garfinkel et al., 2015). How interoception is associated with extroception, proprioception, and motor efferent remains an intriguing open question.

## Data Availability

The datasets presented in this study can be found in the online repositories. https://osf.io/uw3nz/.
