# Supplementary material for: Influence of interoception and body movement on the rubber hand illusion
Source: Front Psychol. 2024 Dec 11;15:1458726. doi: 10.3389/fpsyg.2024.1458726 (PMC11669270; doi:10.3389/fpsyg.2024.1458726)
Supplement: Supplementary file 1 [file Presentation_1.pdf]

Supplementary Material

Table S1.

Japanese BPQ-BA-VSF（Appendix 1 in Kobayashi et al., 2021, https://doi.org/10.4992/jjpsy.85.13235, copied with permission. The original English statements are shown to the right (selected from Table 2 of Cabrera et al., 2017, https://doi.org/10.1002/mpr.1596, copied with permission) .

	Statement	(original)
1	口のなかが乾いているか	My mouth being dry
2	どれだけ早く呼吸をしているか	How fast I am breathing
3	体や身体の一部の腫れ	A swelling of my body or parts of my body
4	腕や足の筋肉の緊張	Muscle tension in my arms and legs
5	水の摂取によるむくみ感	A bloated feeling because of water retention
6	鳥肌	Goose bumps
7	胃や腸の痛み	Stomach and gut pains
8	胃の膨満感や満腹感	Stomach distension or bloatedness
9	くちびるの震え	Tremor in my lips
10	“背筋がぞっとして” 髪の毛が逆立つ感覚	The hair on the back of my neck “standing” up”
11	つばを飲み込む衝動	An urge to swallow
12	どれだけ強く心臓が鼓動しているか	How hard my heart is beating

Table S2.

RHI questionnaire in Japanese. The statements were translated from Table 1 of Berger and Ehrsson, 2023; https://doi.org/10.1007/s00221-023-06586-w, CC BY 4.0), as shown to the right.

	Type	Statement	(original)
1	SBO	偽物の手が自分の手であるかのように感じた	I felt as if the rubber hand were my hand
2	SBO	自分の手を見ているかのように感じた	I felt as if I was looking at my own hand
3	SBO control	自分の手がゴムになっていくように感じた	I felt as if my (real) hand were turning “rubbery”
4	SBO control	自分の手がなくなってしまうかのように感じた	I felt as if I no longer had a right hand; as if my right hand had disappeared
5	SA	偽物の手の動きを制御できるかのように感じた	I felt as if I could control the movements of the rubber hand
6	SA	偽物の手は，まるで私の意志に従うかのように，私が望んだように動いた	The rubber hand moved like I wanted it to, as if it were obeying my will
7	SA control	偽物の手が私を制御しているかのように感じた	I felt as if the rubber hand were controlling me
8	SA control	偽物の手自身が意志をもっているかのように感じた	I felt as if the rubber hand had a will of its own

SBO: sense of body ownership, SA: sense of agency

Table S3.

Descriptive statistics of the IAcc index for each group, including the two outliers.

Group	Mean	SD	Median	q25	q75	Min	Max
Classical							
High IAcc	0.793	0.091	0.783	0.749	0.849	0.664	0.947
Low IAcc	0.390	0.280	0.542	0.140	0.621	0.000	0.647
Moving							
High IAcc	0.787	0.118	0.822	0.681	0.861	0.632	0.946
Low IAcc	0.450	0.205	0.541	0.354	0.609	0.014	0.624

Table S4.

Descriptive statistics of the IS index for each group, including the two outliers.

Group	Mean	SD	Median	q25	q75	Min	Max
Classical							
High IS	27.4	6.65	27.0	21.8	33.5	19	36
Low IS	11.6	4.50	12.5	7.3	15.8	6	17
Moving							
High IS	29.3	9.20	26.0	21.3	34.8	21	45
Low IS	14.2	3.82	14.5	13.3	17.3	8	19

Table S5.

Statistical values of Wilcoxon's signed rank tests on the responses between synchronous and asynchronous trials for each questionnaire item (outliers excluded).

	Classical				Moving (active)				Moving (passive)		
	V	corrected *p*			V	corrected *p*			V	corrected *p*	
item1	162.5	0.004	**		117.5	0.005	**		132.5	0.004	**
item2	141	0.014	*		91	0.004	**		120	0.001	**
item3	62	1.000			73	0.598			85	0.055	+
item4	123.5	0.030	*		43	1.000			42	1.000	
item5	166	0.002	**		133	0.004	**		70	0.156	
item6	62.5	0.659			113	0.020	*		57.5	1.000	
item7	69.5	1.000			38.5	1.000			65.5	1.000	
item8	67	1.000			13.5	0.067	+		33.5	1.000	

Table S6.

Descriptive statistics of the proprioceptive drift (PD) scores for each group (outliers excluded).

Quantiles	*Synchronous*	*Asynchronous*	*PD Index*
Classical			
Median	**0.58**	**-0.05**	**0.78**
25	-0.13	-0.44	0.00
75	2.51	0.66	1.58
Moving (active)			
Median	**0.25**	**0.40**	**0.10**
25	-0.78	-0.53	-1.23
75	0.68	0.80	0.38
Moving (passive)			
Median	**-0.10**	**-0.10**	**0.25**
25	-0.58	-1.13	-0.65
75	0.98	0.58	0.75

Table S7.

Summary of the questionnaire ratings. Same as Table 1, but the outliers are included.

SBO: sense of body ownership, SA: sense of agency.

	Synchronous					Asynchronous			
Quantiles	SBO	SBO control	SA	SA control		SBO	SBO control	SA	SA control
Classical									
Median	**1.63**	**-1.13**	**-1.25**	**-1.25**		**0.00**	**-2.13**	**-2.50**	**-1.75**
P25	1.00	-2.06	-2.06	-2.38		-1.75	-3.00	-3.00	-2.75
P75	2.56	1.06	-0.69	-0.94		1.06	0.06	-1.69	-1.00
Moving (active)									
Median	**1.00**	**-1.00**	**2.00**	**-1.00**		**-1.25**	**-2.25**	**-1.00**	**-0.50**
P25	0.38	-2.50	1.00	-2.50		-2.13	-2.50	-2.13	-1.50
P75	1.75	0.00	2.50	-0.50		-0.50	-0.88	1.13	1.50
Moving (passive)									
Median	**1.00**	**-0.75**	**-0.75**	**-1.00**		**-2.00**	**-2.00**	**-2.00**	**-0.75**
P25	-0.13	-2.50	-1.63	-2.00		-3.00	-3.00	-2.50	-1.25
P75	2.50	0.13	0.00	0.00		-0.88	-0.50	-0.50	-0.50

Table S8. ANOVA tables for moving RHI (outliers excluded).

SBO x IAcc

Source	SS	df	MS	F-ratio	p-value		eta^2
IAcc_group	3.4031	1	3.4031	0.6470	0.4330	ns	0.0269
s x IAcc_group	84.1594	16	5.2600				
mode	1.2920	1	1.2920	0.6142	0.4447	ns	0.0102
IAcc_group x mode	4.1253	1	4.1253	1.9610	0.1805	ns	0.0326
s x IAcc_group x mode	33.6594	16	2.1037				
Total	126.1875	35	3.6054				

SBO x IS

Source	SS	df	MS	F-ratio	p-value		eta^2
IS_group	20.5031	1	20.5031	4.8919	0.0419	*	0.1621
s x IS_group	67.0594	16	4.1912				
mode	1.1281	1	1.1281	0.5023	0.4887	ns	0.0089
IS_group x mode	1.8503	1	1.8503	0.8239	0.3775	ns	0.0146
s x IS_group x mode	35.9344	16	2.2459				
Total	126.1875	35	3.6054				

SA x IAcc

Source	SS	df	MS	F-ratio	p-value		eta^2
IAcc_group	0.4753	1	0.4753	0.1014	0.7543	ns	0.0036
s x IAcc_group	74.9969	16	4.6873				
mode	16.6531	1	16.6531	9.5684	0.0070	**	0.1258
IAcc_group x mode	12.4031	1	12.4031	7.1265	0.0168	*	0.0937
s x IAcc_group x mode	27.8469	16	1.7404				
Total	135.9722	35	3.8849				

Simple main effects

Source	SS	df	MS	F-ratio	p-value		eta^2
IAcc_group at active	4.0111	1	4.0111	1.0171	0.3282	ns	0.0598
Er at active	63.1000	16	3.9437				
IAcc_group at passive	8.8674	1	8.8674	3.5698	0.0771	+	0.1824
Er at passive	39.7437	16	2.4840				
mode at High	0.1406	1	0.1406	0.0805	0.7849	ns	0.0031
s x mode at High	12.2344	7	1.7478				
mode at Low	32.5125	1	32.5125	18.7422	0.0019	**	0.3587
s x mode at Low	15.6125	9	1.7347				

SA x IS

Source	SS	df	MS	F-ratio	p-value	eta^2
IS_group	7.1003	1	7.1003	1.6616	0.2157 ns	0.0528
s x IS_group	68.3719	16	4.2732			
mode	18.8503	1	18.8503	7.7590	0.0132 *	0.1401
IS_group x mode	1.3781	1	1.3781	0.5672	0.4623 ns	0.0102
s x IS_group x mode	38.8719	16	2.4295			
Total	135.9722	35	3.8849			

PD x IAcc

Source	SS	df	MS	F-ratio	p-value	p.eta^2
IAcc_group	1.7405	1	1.7405	0.8746	0.3636 ns	0.0518
s x IAcc_group	31.8395	16	1.9900			
mode	3.3347	1	3.3347	1.4075	0.2528 ns	0.0809
IAcc_group x mode	0.3125	1	0.3125	0.1319	0.7212 ns	0.0082
s x IAcc_group x mode	37.9075	16	2.3692			
Total	75.4100	35	2.1546			

+p < .10, *p < .05, **p < .01, ***p < .001

PD x IS

Source	SS	df	MS	F-ratio	p-value	p.eta^2
IS_group	0.3511	1	0.3511	0.1691	0.6864 ns	0.0105
s x IS_group	33.2289	16	2.0768			
mode	3.1337	1	3.1337	1.3517	0.2620 ns	0.0779
IS_group x mode	1.1281	1	1.1281	0.4866	0.4954 ns	0.0295
s x IS_group x mode	37.0919	16	2.3182			
Total	75.4100	35	2.1546			

+p < .10, *p < .05, **p < .01, ***p < .001
mode: movement type (passive/active)

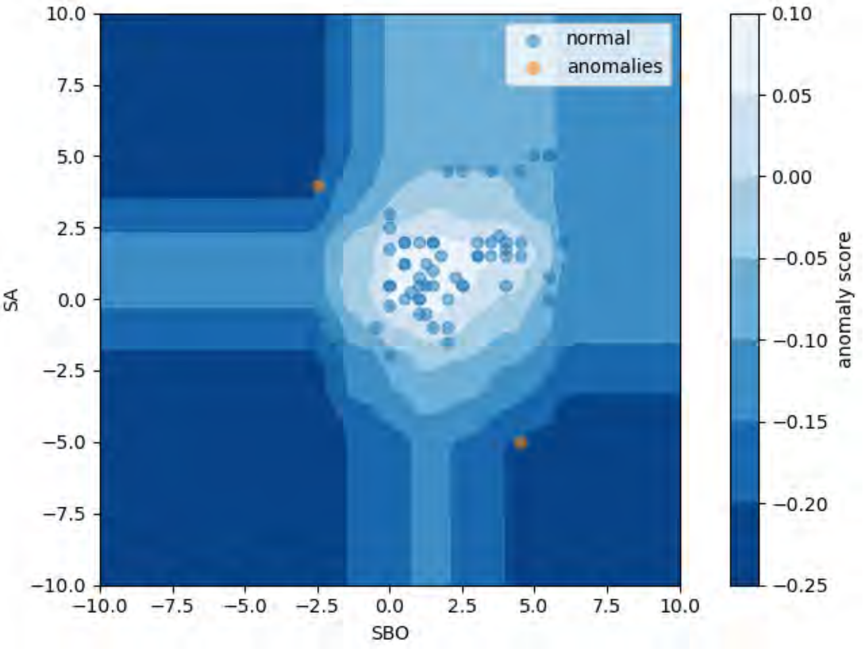

Figure S1

The result of Isolation Forest analysis (Liu et al. 2008) on the SBO and SA scores. Each point represents a pair of SBO and SA scores under all cRHI and mRHI conditions (hence showing 60 points with some overlaps). The threshold for anomaly was set as -0.2, by visual inspection of the plot. The two lowest scores (i.e. highest anomaly) were -0.212 and -0.210, while the third score was -0.148.

We used a python code that was adapted from the website by asmsuechan and yokoponzoo https://kagglenote.com/ml-tips/isolationforest/ (in Japanese, last modified Oct 9, 2023), using the scikit-learn tool (https://scikit-learn.org/1.5/modules/generated/sklearn.ensemble.IsolationForest.html).

Our code and data will be available at our OSF page https://osf.io/uw3nz/ .

Liu, F. T., Ting, K. M., & Zhou, Z. H. (2008). Isolation forest. In *2008 eighth IEEE international conference on data mining* (pp. 413-422). IEEE. https://doi.org/10.1109/ICDM.2008.17

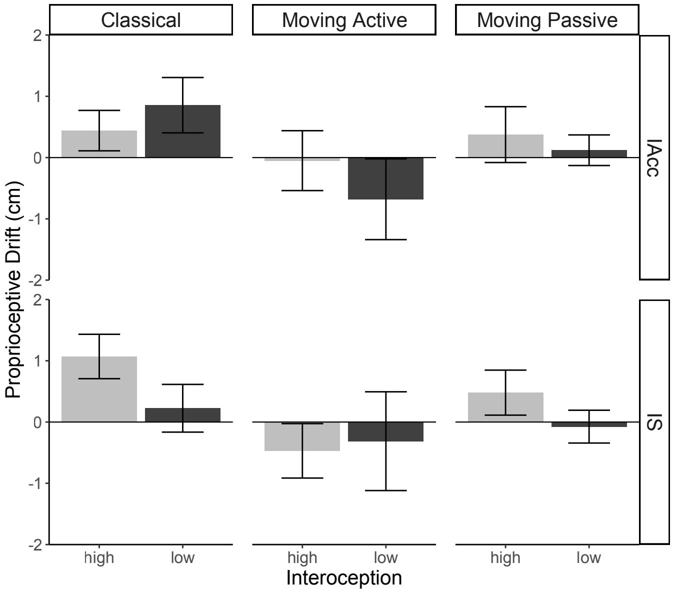

Figure S2

Proprioceptive Drift Index for high and low interoceptive groups.

Statistical tests showed no significant differences between high- and low-groups in any condition.

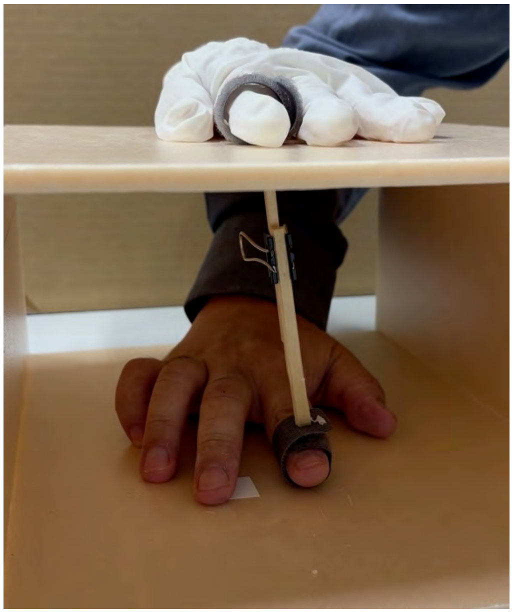

Active Sync (Video 1.mov)

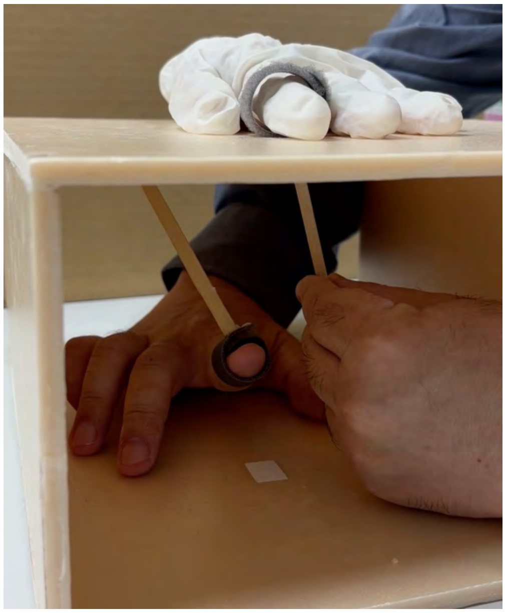

Active Async (Video 2.mov)

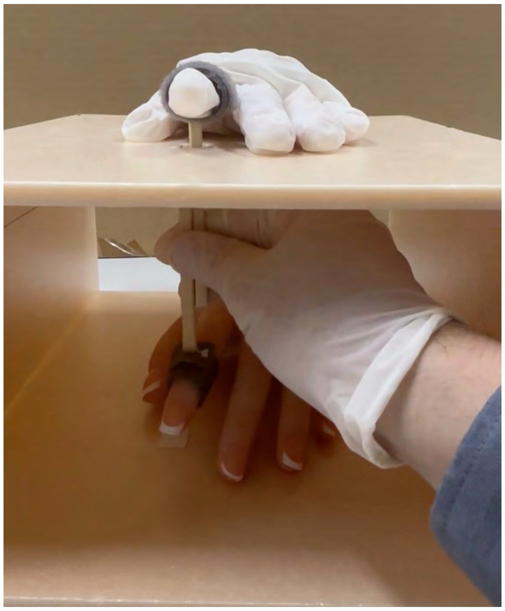

Passive Sync (Video 3.mov)

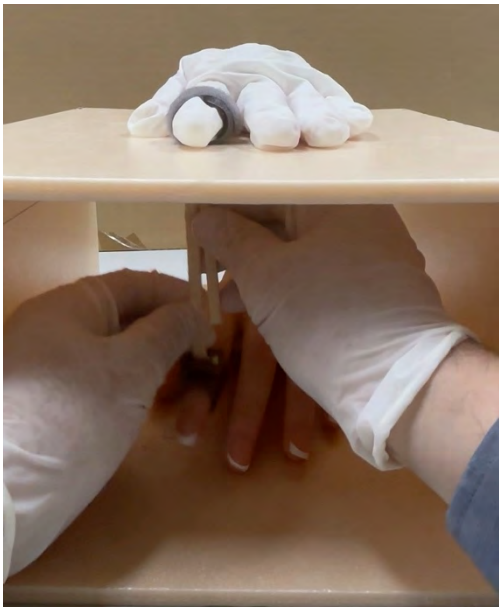

Passive Async (Video 4.mov)

Figure S3

These are still pictures of the movies that show how the real and fake fingers were controlled. Note that the movies were reproduced for demonstration purposes, and details were not the same as in the real experiments.

Movies are provided as separate files.
